# Prevalence and short-term outcomes of primary metabolic and bariatric surgery in non-binary patients: an analysis of the MBSAQIP database

**DOI:** 10.1007/s00464-026-13145-1

**Published:** 2026-08-04

**Authors:** P. Kachornvitaya, M. Wills, J. Barajas-Gamboa, X. Zhu, Y. Lee, S. Navarrete, R. Corcelles, A. Strong, S. Udomsawaengsup, M. Kroh, J. Dang, V. Mocanu

**Affiliations:** 1 Cleveland Clinic Main Campus Hospital, Cleveland, OH USA; 2 Digestive Diseases Institute, Cleveland Clinic Abu Dhabi, Abu Dhabi, United Arab Emirates; 3 Department of Surgery, Faculty of Medicine, Chulalongkorn University, Bangkok, Thailand; 4 Treatment of Obesity and Metabolic Disease Research Unit, Faculty of Medicine, Chulalongkorn University, Bangkok, Thailand

**Keywords:** Metabolic and bariatric surgery, Non-binary, Gender identity, MBSAQIP, Postoperative outcomes

## Abstract

**Introduction:**

Non-binary individuals represent a growing demographic in the U.S. and experience higher rates of obesity. Metabolic and bariatric surgery (MBS) is the most effective treatment for patients with severe and complex obesity, yet data on its use and outcomes in non-binary patients have not yet been studied. Existing research has focused on binary gender categories, leaving a critical gap in understanding surgical outcomes for non-binary individuals. This study compared perioperative characteristics and 30-day outcomes of MBS between non-binary and binary patients.

**Methods:**

A retrospective analysis of the 2020–2023 MBSAQIP database included adult patients undergoing primary sleeve gastrectomy (SG) and Roux-en-Y gastric bypass (RYGB), stratified by gender identity. Baseline demographics, comorbidities, operative details, and 30-day outcomes were compared. Multivariable logistic regression was used to identify predictors of serious postoperative complications.

**Results:**

Among 692,615 patients, 272 (0.04%) identified as non-binary. Their numbers more than doubled over the study period. Compared to binary cohort, non-binary cohort were younger (37.3 ± 11.1 vs. 43.1 ± 11.8 years, *p* < 0.0001), had higher BMI (46.7 ± 8.6 vs. 45.1 ± 7.7 kg/m^2^, *p* = 0.001), and had higher proportions of obstructive sleep apnea (45.6% vs. 37.2%, *p* = 0.004). They underwent RYGB more often (32.7% vs. 27.2%, *p* = 0.040), had longer operative times (91.2 ± 49.2 vs. 84.4 ± 49.2 min, *p* = 0.004), and higher 30-day readmission rates (6.3% vs. 2.9%, *p* = 0.001). No significant differences were found in rates of reoperation, leak, bleeding, VTE, or serious complications. Non-binary identity was not an independent predictor of serious complications (OR 0.98, 95% CI 0.46–2.08, *p* = 0.958). In contrast, non-binary identity independently predicted higher 30-day readmission rates (OR 2.19, 95% CI 1.33–3.59; *p* = 0.002).

**Conclusions:**

Non-binary patients represented a small but increasing proportion of patients undergoing primary MBS. In this 30-day MBSAQIP analysis, non-binary status was independently associated with higher odds of readmission but was not associated with increased adjusted odds of serious complications. Given the small non-binary cohort and limited readmission-detail availability, these findings should be interpreted cautiously and support the need for more nuanced gender data collection and long-term outcomes research.

Non-binary individuals, whose gender identity lies outside traditional binary categories of male and female, represent a small but growing demographic in the U.S., estimated at approximately 1.2 million people, with increasing visibility in healthcare research and clinical care [1,2]. Individuals identifying as non-binary face disproportionately high rates of obesity and metabolic disease, partially due to structural discrimination and psychological distress which may exacerbate unhealthy weight patterns and complicate engagement with preventive health services [3,4]. Metabolic and bariatric surgery (MBS) is widely recognized as the most effective intervention for achieving sustained weight loss and improving obesity-related comorbidities such as type 2 diabetes, hypertension, and obstructive sleep apnea [5]. However, nearly all existing research in bariatric outcomes has focused on binary gender populations, leaving the safety, efficacy, and perioperative profile of surgery in non-binary patients largely unknown.

Importantly, obesity can also act as a barrier to gender-affirming medical and surgical care. Many non-binary and transgender individuals are required to meet strict body mass index (BMI) thresholds to qualify for gender-affirming procedures such as chest reconstruction or genital surgery. For those living with obesity, these BMI restrictions can delay or prevent access to medically necessary care, compounding existing disparities in health equity [6]. To date, no large-scale, representative studies have evaluated short-term outcomes of MBS in non-binary individuals.

To address this knowledge gap, we conducted a retrospective analysis of the MBSAQIP database from 2020 to 2023 to compare baseline characteristics, perioperative factors, and 30-day postoperative outcomes between non-binary and binary patients undergoing primary MBS. We also evaluated whether non-binary identity is independently associated with serious postoperative complications. This study aims to inform more inclusive, evidence-based surgical care for gender-diverse populations.

## Materials and methods

### Data source and ethical approvals

A retrospective analysis was conducted using the Metabolic and Bariatric Surgery Accreditation and Quality Improvement Program (MBSAQIP) Participant Use File (2020–2023), which includes prospectively collected data from nearly 1,000 accredited bariatric centers in the U.S. and Canada. The dataset captures standardized pre-, intra-, and postoperative variables for MBS. Institutional Review Board approval was not required due to the de-identified nature of the dataset.

### Patient variables and population

Adult patients undergoing primary MBS, including sleeve gastrectomy (SG) and Roux-en-Y gastric bypass (RYGB), were included. The cohorts were stratified by gender identity into non-binary and binary cohorts. SG was identified using CPT code 43,775; RYGB was identified using CPT code 43,644; All revisional, conversion, and multi-stage procedures were excluded.

Demographic data (age, sex, race, BMI, smoking status, functional status, ASA class) and comorbidities were extracted and categorized by system: metabolic (e.g., diabetes, hypertension), cardiovascular, pulmonary, renal, and others (e.g., GERD, VTE history, anticoagulant use). All variables followed MBSAQIP definitions. The number of operations performed in each operative year was also reported for both groups.

### Objectives

The primary objectives were to compare perioperative characteristics, trends in delivery over time, and 30-day outcomes of MBS between non-binary and binary cohorts. Secondary objectives were to identify if non-binary gender status was independently associated with adverse 30-day rates of serious complications and readmission using multivariable logistic regression analysis.

Serious 30-day complications were defined as a composite outcome including anastomotic leak, bleeding, cardiac events, cerebrovascular accident, pneumonia, bowel obstruction, unplanned intubation, acute renal failure, venous thromboembolism, deep surgical site infection, wound disruption, sepsis, or death. This composite outcome was designed to capture clinically significant morbidity that would impact patient recovery and healthcare resource utilization, consistent with previous MBSAQIP analyses.

### Statistical analysis

Categorical variables were summarized as absolute values and percentages, and continuous variables as means ± standard deviation. Between-group differences were evaluated using chi-square and ANOVA statistical tests as appropriate. Univariate and multivariable logistic regression identified predictors of serious complications and mortality. Variables with *p* < 0.1 in univariate analysis were included in multivariable models or where deemed clinically relevant. Model performance was evaluated using area under the receiver operating characteristic (AUROC) curve (acceptable if > 0.6) and Brier score (< 0.1 indicates good calibration). A two-sided *p* value < 0.05 was considered statistically significant. All analyses were performed using STATA 17 (StataCorp, College Station, TX).

## Results

### Patient baseline characteristics

A total of 692,615 patients underwent primary MBS; of these, only 272 (0.04%) identified as non-binary, 568,568 (82.1%) as female, and 123,775 (17.9%) as male (Table 1). Compared to binary patients, non-binary patients were significantly younger (mean age 37.3 vs. 43.1 years, *p* < 0.0001) and had a higher mean BMI (46.7 vs. 45.1 kg/m^2^, *p* = 0.001). Racial distribution also differed, with non-binary patients more likely to identify as “Other” (32.0% vs. 16.0%) and less likely to be White (50.7% vs. 63.8%) or Black (17.3% vs. 20.2%) (*p* < 0.0001 for race). Obstructive sleep apnea was more prevalent in non-binary patients (45.6% vs. 37.2%, *p* = 0.004), while hypertension (37.1% vs. 43.8%, *p* = 0.028) and hyperlipidemia (17.3% vs. 22.6%, *p* = 0.035) were less common. No significant differences were observed in diabetes, COPD, immunosuppression, renal insufficiency, or history of thromboembolic or cardiac events.

**Table 1:** Baseline characteristics of the patients

	Non-binary (*n* = 272)	Binary (*n* = 692,343)	*p* value
Age (years, mean ± SD)	37.3 ± 11.1	43.1 ± 11.8	< 0.0001
Gender identity			< 0.0001
Binary			
Female	–	568,568 (82.1)	
Male	–	123,775 (17.9)	
Non-binary	272 (100)	–	
Race			< 0.0001
White	138 (50.7)	441,790 (63.8)	
Black or African American	47 (17.3)	139,562 (20.2)	
Other	87 (32.0)	110,991 (16.0)	
Body mass index (kg/m2, mean ± SD)	46.7 ± 8.6	45.1 ± 7.7	0.001
Functional status			0.937
Independent	271 (99.6)	688,289 (99.5)	
Partially dependent	1 (0.4)	3,312 (0.4)	
Dependent	0 (0.0)	149 (0.1)	
ASA category			0.300
ASA 1–2	40 (14.7)	126,749 (18.3)	
ASA 3	222 (81.6)	541,306 (78.3)	
ASA 4–5	10 (3.7)	23,380 (3.4)	
Smoker in the past year	24 (8.8)	44,569 (6.4)	0.109
Diabetes			0.353
None or diet controlled	212 (77.9)	532,056 (76.8)	
Non-insulin dependent	49 (18.0)	118,028 (17.1)	
Insulin dependent	11 (4.1)	42,259 (6.1)	
Hypertension	101 (37.1)	302,956 (43.8)	0.028
Gastroesophageal reflux disease	89 (32.7)	207,001 (29.9)	0.309
Hyperlipidemia	47 (17.3)	156,641 (22.6)	0.035
Obstructive sleep apnea	124 (45.6)	257,826 (37.2)	0.004
Chronic obstructive pulmonary disease	3 (1.1)	7,850 (1.1)	0.962
Immunosuppressive therapy	8 (2.9)	15,706 (2.3)	0.456
Renal Insufficiency	0 (0.0)	3,642 (0.5)	0.230
History of venous thromboembolism	8 (2.9)	17,795 (2.6)	0.699
Venous stasis	1 (0.4)	5,203 (0.8)	0.464
Therapeutic anticoagulation use	5 (1.8)	19,847 (2.9)	0.309
History of myocardial infarction	3 (1.1)	6,853 (1.0)	0.851
Previous PCI	2 (0.7)	8,477 (1.2)	0.463
Previous major cardiac surgery	1 (0.4)	5,377 (0.8)	0.442

*ASA* American Society of Anesthesiologists, *PCI* percutaneous coronary intervention

The annual number of non-binary patients more than doubled over the study period, increasing from 43 in 2020 (0.03%) to 92 in 2023 (0.05%) (Table 2).

**Table 2:** Annual distribution of metabolic and bariatric surgery in non-binary and binary patients from 2020 to 2023

Operative year	Non-binary	Binary	Total
2020	43 (0.03)	141,429 (99.7)	141,472
2021	65 (0.04)	176,673 (99.6)	176,738
2022	72 (0.04)	193,775 (99.6)	193,847
2023	92 (0.05)	180,466 (99.5)	180,558

### Procedural details and postoperative complications

Non-binary patients were more likely to undergo RYGB compared to binary patients (32.7% vs. 27.2%, *p* = 0.040) and had slightly longer operative times (91.2 ± 49.2 vs. 84.4 ± 49.2 min, *p* = 0.004) (Table 3). Length of stay was marginally longer for non-binary patients (1.4 ± 1.1 vs. 1.3 ± 1.1 days, *p* = 0.052), but the difference was not statistically significant.

**Table 3:** Perioperative details and 30-day postoperative complications

	Non-binary (*n* = 272)	Binary (*n* = 692,343)	*p* value
Operative details			
RYGB, n (%)	89 (32.7)	188,135 (27.2)	0.040
Laparoscopic approach (*n*, %)	272 (100)	692,253 (99.9)	< 0.0001
Operative time (mins), mean ± SD	91.2 ± 49.2	84.4 ± 49.2	0.004
Length of stay (days), mean ± SD	1.4 ± 1.1	1.3 ± 1.1	0.052
Drain placement, n (%)	17 (6.3)	44,694 (6.5)	0.890
Albumin level (g/dL), mean ± SD	4.1 ± 0.5	4.2 ± 0.4	0.001
Surgical complications, *n* (%)			
Leaks	0 (0.0)	1,486 (0.2)	0.444
Postoperative bleeding	2 (0.7)	6,077 (0.9)	0.801
Reoperation	1 (0.4)	6,223 (0.9)	0.353
Non-operative intervention	2 (0.7)	4,695 (0.7)	0.909
Readmission	17 (6.3)	19,988 (2.9)	0.001
Bowel obstruction	0 (0)	1,917 (0.3)	0.385
Wound disruption	0 (0)	290 (0.1)	0.736
Infectious complications, *n* (%)			
Deep SSI	0 (0)	2,026 (0.3)	0.372
Pneumonia	1 (0.4)	1,235 (0.2)	0.460
Sepsis	0 (0)	618 (0.1)	0.622
Medical complications, *n* (%)			
Unplanned intubation	1 (0.4)	589 (0.1)	0.110
Venous thromboembolism	1 (0.4)	2,484 (0.4)	0.980
Acute renal failure	1 (0.4)	313 (0.1)	0.013
Cardiac events	1 (0.4)	765 (0.1)	0.202
Cerebrovascular accidents	0 (0)	81 (0.01)	0.858
Composite outcomes, *n* (%)			
Serious complications	7 (2.6)	18,675 (2.7)	0.900
Mortality	1 (0.4)	477 (0.1)	0.061

*RYGB* Roux-en-Y gastric bypass, *SSI* superficial site infection

The overall 30-day postoperative complication rates were low and comparable between binary and non-binary groups. Non-binary patients experienced significantly higher 30-day readmission rates (6.3% vs. 2.9%, *p* = 0.001), but there were no differences in reoperation, leaks, postoperative bleeding, or need for non-operative interventions. Similarly, infectious (deep SSI, pneumonia, sepsis) and medical complications (VTE, cardiac events) occurred at similar rates in both cohorts. The most commonly reported reasons for readmission included abdominal pain, nausea and vomiting, and dehydration. Although readmission was more frequent among non-binary patients, readmission diagnoses were not captured for more than 90% of readmission records in the analyzed dataset. Therefore, the specific clinical indications for readmission could not be reliably characterized. The overall rate of serious complications was not significantly different (2.6% vs. 2.7%, *p* = 0.900). Mortality was rare and not statistically different (0.4% vs. 0.1%, *p* = 0.061).

### Multivariable logistic regression for predictors of serious complications and 30-day readmission

In multivariable logistic regression (Table 4), non-binary gender identity was not independently associated with serious postoperative complications (adjusted OR 0.98, 95% CI 0.46–2.08, *p* = 0.958). Independent predictors of serious complications included higher BMI (per 5 kg/m^2^, OR 1.01, *p* = 0.021), older age (per 10 years, OR 1.04, *p* < 0.001), longer operative time (per 30 min, OR 1.10, *p* < 0.001), insulin-dependent diabetes (OR 1.16, *p* < 0.001), hypertension (OR 1.21, *p* < 0.001), renal insufficiency (OR 2.09, *p* < 0.001), history of myocardial infarction (OR 1.50, *p* < 0.001), therapeutic anticoagulation use (OR 2.00, *p* < 0.001), and undergoing RYGB (OR 1.86, *p* < 0.001).

**Table 4:** Predict risk factors for serious complications

	Adjusted odds ratio^*^	95% confidence interval	*p* value
Higher BMI (per 5 kg/m^2^)	1.01	1.00–1.02	0.021
Older age (per 10 years)	1.04	1.03–1.05	< 0.001
Longer operative time (per 30 min)	1.10	1.09–1.11	< 0.001
Non-binary identity	0.98	0.46–2.08	0.958
Diabetes			
Non-insulin dependent	1.00	0.96–1.04	0.915
Insulin dependent	1.16	1.10–1.23	< 0.001
Hypertension	1.21	1.17–1.25	< 0.001
Renal insufficiency	2.09	1.84–2.37	< 0.001
History of myocardial infarction	1.50	1.35–1.66	< 0.001
Therapeutic anticoagulant	2.00	1.88–2.13	< 0.001
Robotic-assisted surgery	1.03	0.99–1.06	0.103
RYGB	1.86	1.80–1.92	< 0.001

*BMI* body mass index, *RYGB* Roux-en-Y gastric bypass

AUROC = 0.65; Brier score = 0.03

In a separate model examining predictors of 30-day readmission (Table 5), non-binary gender identity was independently associated with increased odds of readmission (adjusted OR 2.19, 95% CI 1.33–3.59, *p* = 0.002). Other significant predictors of readmission included longer operative time (OR 1.10 per 30 min, 95% CI 1.09–1.11, *p* < 0.001), insulin-dependent diabetes (OR 1.41, 95% CI 1.34–1.48, *p* < 0.001), hypertension (OR 1.18, 95% CI 1.14–1.21, *p* < 0.001), renal insufficiency (OR 2.03, 95% CI 1.80–2.31, *p* < 0.001), history of myocardial infarction (OR 1.34, 95% CI 1.20–1.49, *p* < 0.001), therapeutic anticoagulation (OR 2.14, 95% CI 2.01–2.27, *p* < 0.001), robotic-assisted surgery (OR 1.07, 95% CI 1.03–1.10, *p* < 0.001), and undergoing RYGB (OR 1.78, 95% CI 1.72–1.84, *p* < 0.001).

**Table 5:** Predict risk factors for 30-day readmission

	Adjusted odds ratio^*^	95% confidence interval	*p* value
Higher BMI (per 5 kg/m^2^)	1.01	1.00–1.02	0.121
Older age (per 10 years)	0.99	0.98–1.00	0.001
Longer operative time (per 30 min)	1.10	1.09–1.11	< 0.001
Non-binary identity	2.19	1.33–3.59	0.002
Diabetes			
Non-insulin dependent	0.97	0.93–1.01	0.123
Insulin dependent	1.41	1.34–1.48	< 0.001
Hypertension	1.18	1.14–1.21	< 0.001
Renal insufficiency	2.03	1.80–2.31	< 0.001
History of myocardial infarction	1.34	1.20–1.49	< 0.001
Therapeutic anticoagulant	2.14	2.01–2.27	< 0.001
Robotic-assisted surgery	1.07	1.03–1.10	< 0.001
RYGB	1.78	1.72–1.84	< 0.001

*BMI* body mass index, *RYGB* Roux-en-Y gastric bypass

AUROC = 0.64; Brier score = 0.03

## Discussion

To our knowledge, this represents the first study to characterize primary MBS outcomes in patients who self-identify as non-binary within a large standardized multicentered international clinical dataset. Our study found that non-binary patients were younger and had higher BMI with a greater metabolic comorbidity burden. Although absolute 30-day readmission rates were low, the risk of readmission was more than doubled compared with binary patients, and non-binary identity remained independently associated with 30-day readmission with no associated increased odds of serious complications.

Despite limited literature, prior work has highlighted that gender minority bariatric patients are underrepresented in bariatric datasets and may face barriers related to healthcare access, documentation of gender identity, and culturally competent care [7]. These patterns are mirrored in other surgical fields. Awe et al. reported that LGBTQ + patients often experience higher perioperative risk and worse postoperative outcomes due to psychosocial burden, healthcare avoidance, and lack of culturally competent care environments [8]. Similarly, Pratt-Chapman et al. found that sexual and gender minority cancer survivors report lower satisfaction with care, higher psychological distress, and reduced engagement in follow-up, all factors that plausibly contribute to increased postoperative care utilization such as readmissions [9].

In our cohort, non-binary patients were significantly younger, consistent with prior reports indicating that 76% of non-binary adults are between 18 and 29 years of age [1], and had a higher preoperative BMI. Additionally, a greater proportion of non-binary patients underwent RYGB compared to binary patients, which may be attributable to their higher preoperative BMI. This is consistent with prior studies of transgender individuals, where obesity is more prevalent than in the general population, and weight gain is commonly observed after initiation of gender-affirming hormone therapy [10,11]. Hormonal changes, mental health stressors, and body image dissatisfaction may all contribute to earlier or more urgent pursuit of MBS in gender-diverse individuals [7, 10]. To this end, our study highlights the importance of further clarifying unique barriers faced for those identifying as non-binary with respect to access, delivery, and postoperative care to continue optimizing outcomes.

Notably, non-binary patients in our study had significantly higher 30-day readmission rates, and non-binary status remained independently associated with readmission after adjustment. However, this finding should be interpreted cautiously. MBSAQIP does not provide sufficiently complete information regarding the specific indications for readmission in this cohort, with readmission diagnoses missing in more than 90% of cases. Therefore, the mechanism underlying the higher readmission rate cannot be determined from this dataset, and no causal inference can be made. The absence of increased rates of reoperation, leak, bleeding, or serious complications suggests that the higher readmission rate was not clearly accompanied by a measurable increase in major short-term morbidity. Potential explanations may include differences in outpatient access, postoperative support, communication during discharge planning, or access to affirming primary and surgical care; however, these factors are not captured in MBSAQIP and remain speculative. Future prospective studies incorporating readmission indications, social support, care access, patient-reported experience, and follow-up adherence are needed to clarify whether the observed readmission difference reflects clinical, social, or healthcare-delivery factors.

Additionally, we found no significant difference in rates of serious complications between non-binary and binary patients, and non-binary identity was not an independent predictor of adverse postoperative events. Instead, predictors of complications were more closely associated with patient comorbidities and the type of surgical procedure performed. These findings are consistent with data from Rahimi et al., who found that after adjusting for demographic and clinical factors, gender itself (male vs. female) was not a significant predictor of serious complications following SG or RYGB [12]. Our findings extend this understanding to non-binary patients, suggesting that with appropriate surgical and medical care, MBS is equally safe across gender identity.

It is important to acknowledge that MBS may also serve as a critical gateway to gender-affirming surgical care for non-binary and transgender individuals who are otherwise ineligible due to BMI restrictions. Many centers performing gender-affirming procedures impose BMI thresholds for operative candidacy. In this context, MBS offers not only metabolic and functional benefits but also facilitates access to gender-affirming interventions that can significantly improve psychological well-being and quality of life. A retrospective study from the Netherlands demonstrated that transgender individuals who underwent MBS prior to gender-affirming surgery experienced substantial weight loss, enabling the safe performance of chest and genital procedures with acceptable complication rates [11]. This underscores the importance of recognizing MBS as part of a broader continuum of care for gender-diverse patients, particularly when used strategically to support transition goals. As MBS may be the first complex surgical commitment with healthcare providers prior to more invasive gender-affirming care, further work is needed from metabolic health centers to optimize bariatric delivery and care for these individuals.

The small number of non-binary patients is the central limitation of this study. Although MBSAQIP provided a large overall cohort, only 272 patients were categorized as non-binary, and rare postoperative events occurred infrequently. Therefore, the study may be underpowered to detect clinically meaningful differences in uncommon outcomes such as leak, venous thromboembolism, mortality, or individual serious complications. The wide confidence interval around the adjusted association between non-binary status and serious complications reflects this limitation. The MBSAQIP database also does not currently include granular data on gender-affirming hormone therapy, history of gender-affirmation surgeries, or validated measures of gender identity beyond administrative fields; these factors may influence health status and perioperative risk. MBSAQIP also lacks several potential confounders relevant to postoperative utilization, including socioeconomic status, insurance type, mental health diagnoses, access to primary care or affirming care, social support, follow-up adherence, center volume, and surgeon experience. Many of these variables are not routinely captured in large surgical registries because of privacy concerns and challenges in standardizing sensitive social and access-to-care measures across institutions. Given the > 90% missingness for causes of readmission, the observed association between non-binary status and readmission is not immediately actionable and should be considered hypothesis-generating rather than explanatory. The dataset cannot determine whether readmissions were driven by surgical complications, dehydration, pain, outpatient access barriers, social support, or other healthcare-delivery factors. Finally, the study is limited to 30-day outcomes. MBSAQIP does not capture long-term weight loss, comorbidity remission, nutritional complications, quality of life, patient satisfaction, or access to subsequent gender-affirming procedures. Therefore, this analysis cannot determine long-term safety, efficacy, or patient-centered benefit of MBS among non-binary patients.

Despite these limitations, our results underscore the need for improved sexual orientation and gender identity (SOGI) data collection in surgical registries and targeted research on gender-diverse populations, particularly within the metabolic field where such research remains sparse [7]. Future studies should further aim to integrate comprehensive SOGI measures, explore the psychosocial determinants of postoperative outcomes, and strive to better assess long-term bariatric efficacy in non-binary and other sexual and gender minority patients.

## Conclusion

Non-binary patients undergoing primary MBS represented a small but increasing proportion of MBSAQIP cases from 2020 to 2023. In this short-term analysis, non-binary patients were younger, had higher BMI, and experienced higher 30-day readmission rates. Given the small non-binary cohort and limited readmission-detail availability, these findings should be interpreted cautiously and support the need for more nuanced gender data collection and long-term outcomes research.
